# Assessing the Beneficial Effects of the Immunomodulatory Glycan LNFPIII on Gut Microbiota and Health in a Mouse Model of Gulf War Illness

**DOI:** 10.3390/ijerph17197081

**Published:** 2020-09-27

**Authors:** Ryan S. Mote, Jessica M. Carpenter, Rachel L. Dockman, Andrew J. Steinberger, Garret Suen, Thomas Norberg, Donald A. Harn, John J. Wagner, Nikolay M. Filipov

**Affiliations:** 1 Department of Physiology and Pharmacology, University of Georgia, Athens, GA 30602, USA; ryan.mote25@uga.edu (R.S.M.); jessica.carpenter@uga.edu (J.M.C.); rld91076@uga.edu (R.L.D.); jwagner@uga.edu (J.J.W.); 2 Department of Bacteriology, University of Wisconsin—Madison, Madison, WI 53706, USA; asteinberger@wisc.edu (A.J.S.); gsuen@wisc.edu (G.S.); 3 Department of Chemistry-BMC, University of Uppsala, 75123 Uppsala, Sweden; thomas.norberg@kemi.uu.se; 4 Department of Infectious Diseases, University of Georgia, Athens, GA 30602, USA; dharn@uga.edu; 5 Center for Tropical and Emerging Infectious Diseases, University of Georgia, Athens, GA 30602, USA

**Keywords:** Gulf War Illness, gut inflammation and health, gut microbiome, lacto-N-fucopentaose-III (LNFPIII), permethrin, pyridostigmine bromide

## Abstract

The microbiota’s influence on host (patho) physiology has gained interest in the context of Gulf War Illness (GWI), a chronic disorder featuring dysregulation of the gut–brain–immune axis. This study examined short- and long-term effects of GWI-related chemicals on gut health and fecal microbiota and the potential benefits of Lacto-N-fucopentaose-III (LNFPIII) treatment in a GWI model. Male C57BL/6J mice were administered pyridostigmine bromide (PB; 0.7 mg/kg) and permethrin (PM; 200 mg/kg) for 10 days with concurrent LNFPIII treatment (35 μg/mouse) in a short-term study (12 days total) and delayed LNFPIII treatment (2×/week) beginning 4 months after 10 days of PB/PM exposure in a long-term study (9 months total). Fecal 16S rRNA sequencing was performed on all samples post-LNFPIII treatment to assess microbiota effects of GWI chemicals and acute/delayed LNFPIII administration. Although PB/PM did not affect species composition on a global scale, it affected specific taxa in both short- and long-term settings. PB/PM elicited more prominent long-term effects, notably, on the abundances of bacteria belonging to *Lachnospiraceae* and *Ruminococcaceae* families and the genus *Allobaculum*. LNFPIII improved a marker of gut health (i.e., decreased lipocalin-2) independent of GWI and, importantly, increased butyrate producers (e.g., *Butyricoccus*, *Ruminococcous*) in PB/PM-treated mice, indicating a positive selection pressure for these bacteria. Multiple operational taxonomic units correlated with aberrant behavior and lipocalin-2 in PB/PM samples; LNFPIII was modulatory. Overall, significant and lasting GWI effects occurred on specific microbiota and LNFPIII treatment was beneficial.

## 1. Introduction

Gulf War Illness (GWI), which afflicts one-third of the veterans from the 1990–1991 Gulf War (GW), is a chronic disorder presenting with a myriad of debilitating symptoms, including neurological, musculoskeletal, immunological and gastrointestinal disturbances that appeared shortly after veterans returned from deployment and have persisted or worsened throughout the last 30 years [1]. Epidemiological evidence suggests that co-exposure to numerous toxicants, including neuro-prophylactics (pyridostigmine bromide: PB), pesticides (permethrin: PM and N,N-Diethyl-methylbenzamide: DEET), and chemical nerve agents (sarin) during the GW may be linked to GWI etiopathogenesis [1]. Previous work suggests GW-related exposures accelerate age-related chronic conditions, such as high blood pressure, increased heart attack and stroke risk, diabetes, and arthritis [2]; many of the persisting symptoms GWI veterans experience (i.e., cognitive, memory and motor impairments) could be exacerbated by accelerated aging [1,2]. Veterans with GWI report more symptoms akin to Parkinson’s Disease (PD) than GW controls [3]. GWI and PD share gastrointestinal (GI) ailments and motor dysfunction in their symptomatology [1,4,5,6]. Moreover, GI disturbances precede PD motor dysfunction, and a recent study suggests that aberrant gut–brain–immune axis plays a role in the neurobehavioral deficits in this disease [7].

Although veterans with GWI experience GI symptoms, such as nausea, diarrhea and abdominal pain/cramps, and GI dysfunction is a prominent GWI diagnostic characteristic [1,5,8,9], studies investigating GI disturbances remain limited. Recent work found that enteric dysbiosis occurs within GWI context [10,11]. It was also suggested that alterations in the gut–brain–immune axis may be involved in GWI, as veterans with GWI and GI ailments have higher levels of circulating tumor necrosis factor receptor 1 (TNF-RI) [11]. Exposing laboratory models to GW chemicals (i.e., PB alone or PB and PM) resulted in alterations in the murine microbiota, gut motility, and inflammatory signaling (e.g., toll-like receptor 4; TLR-4) in both the gut and brain [10,12,13]. While clinical reports of peripheral and central inflammation are, respectively, variable and sparse, a consensus for immune disruption has emerged in GWI etiology, as GWI veterans exhibit increases in circulating cytokines and glial (TSPO) activation compared to controls [14,15,16,17]. Immune alterations are further driven by (neuro) inflammatory increases in multiple laboratory models of GWI; this showcases how interplay between the nervous and immune systems impacts GWI development, specifically regarding the effects of inflammation on neurological and neurobehavioral function [18,19,20,21,22,23,24]. However, the relationship between GWI GI symptomology, immune alterations, and behavioral outcomes have not been investigated.

Interestingly, one clinical study found the only circulating molecule (cytokine-related) significantly affected in GWI veterans was the soluble TNF-RI and that GI symptoms did not influence bacteria found to be significantly different between veterans with GWI and controls [11]. Another recent study found administration of sodium butyrate through oral gavage in a mouse model of GWI restored enteric niacin receptor, tight junction protein, and TLR-4 expression levels to control levels [12]. Additionally, butyrate administration increased levels of bacteria that produce butyrate and have been the focus of intense investigation due to their use in potential probiotics (e.g., *Roseburia* sp., *Bifidobacterium;* [12]). While these bacteria increased in GWI animals treated with butyrate, it is unclear whether these increases are a result of direct butyrate administration, as these bacteria do not utilize butyrate for sustenance or growth. Finally, evidence for the beneficial effects of *Bifidobacterium*, another genus commonly studied as a probiotic, is equivocal as only some strains within this genus may be beneficial for patients with GI disorders [25,26,27,28,29,30]. This suggests that genus, and perhaps, strain specific maladaptation of the GWI microbiota ought to be tested. Overall, the interactions along the gut–brain–immune axis are incredibly complex [31,32], but are worthy of further investigation in the context of GWI pathophysiology and potential treatments.

Cure-all treatments for GWI do not currently exist due, in part, to GWI’s complexity; however, experimental therapeutic interventions have provided benefits to some GWI symptoms [33,34,35,36]. Current treatments for GI disorders in general, include altering diet and/or lifestyle, probiotic supplementation, and fecal microbiota transplants; some have been beneficial to patients with neurological disorders including PD, Multiple Sclerosis, and Alzheimer’s Disease [37,38]. However, explored treatment interventions for gut health in veterans with GWI remain limited [12].

Immunotherapies may be an advantageous treatment option considering the pathogenic role inflammation and immune dysregulation play in GWI symptomology. Lacto-N-fucopentaose III (LNFPIII), a glycan found in human milk that, to date, has had no documented adverse outcomes and has shown promising immunomodulatory effects by reducing peripheral and central inflammation [19,39,40,41,42,43]. When conjugated to a dextran carrier, LNFPIII skews the inflammatory balance of the innate immune system in an anti-inflammatory direction by activating CD14/TLR-4 signaling for extracellular signal-regulated kinase (ERK) dependent production of anti-inflammatory mediators [39,40,41,42,43]. Thus, within the context of GWI, LNFPIII may be beneficial in absolving GWI-induced gut inflammation and subsequent neuroinflammation through its modified anti-inflammatory TLR-4 signaling [10]. In fact, our earlier studies demonstrated LNFPIII’s beneficial effects in preventing and reducing brain wide monoaminergic disbalance and inflammation in the hippocampus after acute experimental GWI exposure [19], as well as restoring long-term behavioral deficits caused by PB/PM exposure, particularly in motor function. Whether LNFPIII modulates gut microbiota and gut health is currently unknown.

This study examines the effects of exposure to GW-related chemicals on GI microbial ecology in an established model of GWI (PB/PM). Our earlier studies using this established exposure paradigm indicated acute neurological [19] and long-term neurobehavioral deficits that were largely restored by the immunotherapeutic, LNFPIII. However, while some data exist on the human and animal GI effects of GWI after exposure, the short- and long-term GI effects of this GWI treatment paradigm have not been investigated. Moreover, while LNFPIII had beneficial effects on neuroinflammation and altering behavioral deficits, there is no published evidence that it is beneficial for targeting GWI GI-related symptoms. Thus, the objectives of the present study were to (i) characterize the short- and long-term implications of PB/PM exposure on gut microbiota, gut motility, and intestinal inflammation, and (ii) correlate gut health to GWI-related neurological aberrations (i.e., motor deficits). Finally, LNFPIII treatment, including when treatment was initiated months after PB/PM exposure ended, was evaluated for its beneficial effects in modulating the aforementioned parameters.

## 2. Materials and Methods

### 2.1. Materials

Pyridostigmine bromide (PB; ≥98% purity; Sigma Aldrich, St. Louis, MO, USA) and permethrin (PM; 29.5% cis/69.5% trans isomer; 99% purity; Chem Service Inc., West Chester, PA, USA) were used for animal treatments in this study. Lacto-N-fucopentaose III (LNFPIII) dextran conjugate was produced as previously described [43]. Carmine red powder (Sigma) and methylcellulose (Sigma) were used for the gut motility test. All additional chemicals and reagents used in this study, unless otherwise noted, were of analytical or higher grade and were obtained from Sigma or Fisher Scientific (Hampton, NH, USA).

### 2.2. Animals

Male C57BL/6J mice (8–9 weeks old; Jackson Laboratories, Bar Harbor, ME, USA) were housed 4 per cage in an environmentally controlled room (22–24 °C) and maintained on a 12 h light/dark cycle (0700–1900 lights on) for one week of acclimation and throughout the study. Mice were handled daily for one week prior to the start of the study to minimize experimenter-induced stress. Food and water were available ad libitum. All procedures were approved in advance by the University of Georgia Institutional Animal Care and Use Committee (initial approval date: 14 April 2016) and were in accordance with the latest National Institutes of Health guidelines.

### 2.3. GWI Model

**Figure 1:**
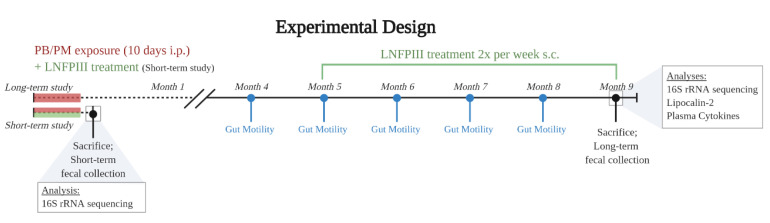
Experimental design. In both the short- and long-term studies, mice were exposed to pyridostigmine bromide (PB) and permethrin (PM) for 10 days for Gulf War Illness (GWI) induction. Lacto-N-fucopentaose-III (LNFPIII) treatment was given concurrently during the short-term study and beginning 4 months post PB/PM exposure in the long-term study. Gut motility was examined monthly from month 4 to 8. Microbiota sequencing of the 16S rRNA gene was performed in both the short- and long-term studies. Additionally, in the long-term study, lipocalin-2 (fecal, plasma) and cytokines (plasma) levels were examined.

The experimental design for this study is shown in Figure 1. Following the Zakirova [18] model in both the short- (12 days; *N* = 24 mice) and long-term (9 months; *N* = 59) studies, mice were randomly divided into treatment groups and treated daily for 10 days with a combination of PB and PM (0.7 and 200 mg/kg, respectively) or DMSO vehicle (i.p.). In the short-term study, the immunomodulatory treatment, LNFPIII, or dextran vehicle (both 35 µg/mouse; s.c.) were administered concurrently with PB/PM as in [19]. The treatment groups in the short-term study were as follows: DMSO-Dextran (*n* = 6), DMSO-LNFPIII (*n* = 6), PB/PM-Dextran (*n* = 6), and PB/PM-LNFPIII (*n* = 6). In the long-term study, four months after the initial PB/PM exposure, mice were randomly subdivided into LNFPIII or vehicle groups and were treated twice a week until study completion with LNFPIII or dextran vehicle (both 35 µg/mouse; s.c.). Thus, from this point onward there were 4 treatment groups: DMSO-Dextran (*n* = 13), DMSO-LNFPIII (*n* = 14), PB/PM-Dextran (*n* = 14), and PB/PM-LNFPIII (*n* = 14).

### 2.4. Gut Motility

The carmine red gut transit test [44] was used to determine gut motility deficits with the modifications described in [45]. This test was performed monthly by administering the dye via oral gavage (6% carmine red in 0.5% methylcellulose; 0.3 mL/mouse) and monitoring the mouse for the first appearance of colored fecal pellet over a 6 h period. For the test, each mouse was single housed and food restricted for 1 h prior to carmine red administration. Once the latency was recorded, each mouse was returned to its home cage.

### 2.5. Lipocalin-2 ELISA

Intestinal inflammation was determined by measuring fecal and plasma levels of Lipocalin-2 (Lcn-2), a protein upregulated in multiple inflammatory diseases including inflammatory bowel disease (IBD), by utilizing methods described in [46]. Briefly, a small sample from previously frozen fecal content was weighed and transferred to a new, sterile polypropylene tube. Samples were reconstituted in 0.1% Tween 20 PBS (100 mg/mL) and vortexed for 25 min until fully homogenized. Samples were then centrifuged for 10 min at 13,200 rcf and 4 °C, supernatants were collected, and Lcn-2 levels were measured. For this test, a Duoset murine Lcn-2 ELISA kit (R&D Systems, Minneapolis, MN, USA) was used per manufacturer’s instructions. Fecal supernatants were run neat and plasma was diluted 1:500 in ELISA reagent diluent prior to analysis. All samples were run in duplicate.

### 2.6. Sample Collection

Following euthanasia, blood (approximately 1 mL) was collected in tubes (Na citrate 0.109 M, 3.2% BD Vacutainer, Becton, Dickinson and Company, San Jose, CA, USA) for plasma harvesting. Immediately afterwards, organs (brain, inguinal lymph nodes, spleen, thymus, liver and kidney) were weighed and frozen on dry ice. Fecal contents were collected from the cecum and weighed under sterile conditions prior to storage placement in a sterile polypropylene tube. All samples were stored at −80 °C until analysis.

### 2.7. Plasma Cytokine Analysis

A Milliplex Cytokine Panel (EMD Millipore Corporation; Billerica, MA, USA) was used to assess plasma concentrations of the following cytokines/chemokines: interferon gamma (IFNγ), macrophage inflammatory protein 3 alpha (MIP-3α/CCL20), interleukin (IL)-1β, IL-22, IL-23, IL-27p70, IL-27, IL-15, IL-17A, Il-17/IL-25, IL-17F, IL-33, IL-31, tumor necrosis factor alpha (TNFα) and beta (TNFβ), IL-4, IL-5, IL-28B, IL-10, IL-13, granulocyte-macrophage colony-stimulating factor (GM-CSF), CD40 ligand (CD40L), and IL-2. Briefly, plasma samples were added to a 96-well plate followed by the addition of premixed, antibody-immobilized beads and incubated with agitation on a shaker at 4 °C overnight. Following washes (3×), detection antibodies were added and incubated for 1 h followed by addition of Streptavdidin-Phycoerythrin for 30 min with agitation on a shaker at room temperature. After final washes (3×), sheath fluid was added, and the data from the plate was collected on a MagPix instrument using xPONENT v.4.2 (Luminex Corp., Austin, TX, USA). Data were analyzed with Milliplex Analyst software, v.5.1 (EMD Millipore, Burlington, MA, USA). Data were extracted based on either a 4- or 5-parameter log curve.

### 2.8. DNA Extraction

Fecal genomic DNA was extracted using commercially available Qiagen (Qiagen; Hilden, Germany) DNeasy PowerSoil Kit (100; Cat. No.: 12888-100) following the manufacturer’s protocols. All extracted DNA samples were resuspended in Tris-EDTA (TE) buffer and quantified using a Qubit^®^ Fluorometer (Invitrogen, San Diego, CA, USA). Extracted DNA samples and TE buffer negative control were then taken through the amplification protocol below.

### 2.9. DNA Amplification and Sequencing

Samples were diluted to 1 ng/µL for amplification, and universal bacterial primers for the 16S rRNA variable region V4, as previously described in detail [47], were used in the amplification reactions. Water was used for PCR negative control. The sequenced controls and samples were taken through quality filtering and normalization procedures described next.

### 2.10. 16S rRNA Fecal Sequence Processing and Bioinformatics Analysis

Raw sequence files were obtained in fastq format and processed using mothur v.1.38.1 [48] as in [49] and modified in [50]. Alpha diversity metrics were tested for effects by exposure to GWI chemicals and LNFPIII by using the non-parametric Kruskal–Wallis test by ranks. A non-parametric permutational analysis of variance (PERMANOVA) was used to test for effects on the entire microbiota community with a 2 *×* 2 factorial design using GWI treatment (±) and LNFPIII (±) exposure as the two factors. Linear discriminant analysis effect size (LefSe) was performed using the Huttenhower lab’s galaxy instance with the relative abundance table as input (https://huttenhower.sph.harvard.edu/galaxy/; [51]); Kruskall–Wallis (*p* < 0.05); Pairwise Wilcoxon (*p* < 0.05); logarithmic Linear discriminant analysis (LDA) score (>2.0). All other statistical microbiota analyses were performed using R [52]. Correlational analyses were performed using the Hmisc R package [53] with Spearman correlation coefficient and significance set to *p* < 0.05. Heatmaps were generated using gplots library [54] and final iterations were edited in Microsoft PowerPoint (Redmond, WA, USA).

### 2.11. Accession Number(s) of DNA Sequences

All DNA sequences are publicly available in the NCBI Sequence Read Archive and are accessible under BioProject accession No. PRJNA665703.

### 2.12. Statistical Analysis for Gut Motility, Lcn-2, and Plasma Cytokines

A two-way analysis of variance (ANOVA) was used to determine main treatment effects or interactions. If an ANOVA was significant (*p* ≤ 0.05), treatment means were separated by Student–Newman–Keuls (SNK) post-hoc test or planned pairwise comparisons (Student’s t-test, as appropriate). All data were analyzed using SigmaPlot 12.5 (San Jose, CA, USA), and all graphs were generated using GraphPad Prism 5 (San Diego, CA, USA). A heat map of the plasma cytokines post-statistical analysis was generated using gplots library in R 3.4.2 software [54].

## 3. Results

### 3.1. Alpha/Beta Diversity Metrics and Global Fecal Microbiota Effects

All samples had an average Good’s coverage of 99.85 ± 0.01% ($\bar{x}$ ± SD; range: 99.59–99.94%), indicating that the sequencing depth captured most of the species diversity. Exposures to PB/PM or LNFPIII had no significant effect (*p* ≥ 0.12) on the global microbiota composition at either the acute or chronic time points when using the Bray–Curtis (abundance) or Jaccard (presence/absence) dissimilarity matrices. In addition, there were no significant main effect (*p* > 0.05) on either Shannon’s diversity or Chao1 richness (Figure S1) profiles of PB/PM or LNFPIII at any time point. However, the fecal microbiota’s Shannon’s diversity 9 months after PB/PM exposure exhibited the following trend (*p* = 0.074): PB/PM-Dextran resulted in an increase, while LNFPIII lowered the diversity index to control levels (Figure S1).

### 3.2. Linear Discriminant Analysis of Effect Size (LEfSe)

**Figure 2:**
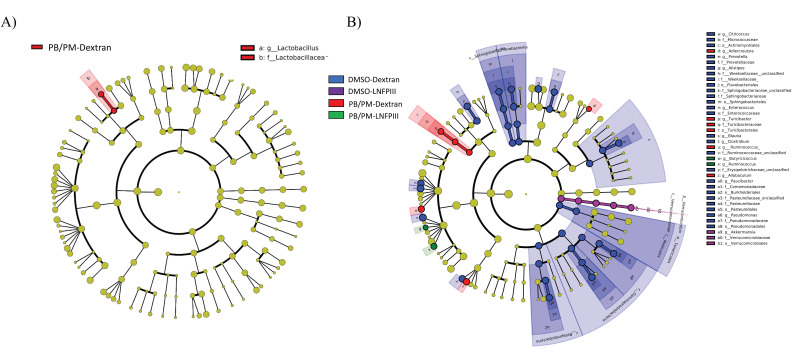
Linear discriminant analysis (LDA) effect size (LEfSe; Kruskall–Wallis (*p* < 0.05); Pairwise Wilcoxon (*p* < 0.05); logarithmic LDA score > 2.0) of the fecal microbiota of mice exposed to either DMSO-Dextran, DMSO-LNFPIII, PB/PM-Dextran, or PB/PM-LNFPIII. Greater sequence abundance for specific taxa at (**A**) 6 h, where red indicates increased abundance in PB/PM-Dextran samples or (**B**) 9 months, where blue, purple, red, and green shading indicates greater abundance in DMSO-Dextran, DMSO-LNFPIII, PB/PM-Dextran, or PB/PM-LNFPIII mice, respectively. Taxonomic rank labels are provided before bacterial names: “p_; c_; o_; f_; g_” indicate phylum, class, order, family, and genus, respectively. Letters and numbers within the cladograms refer to bacterial names located in the key to the right of each cladogram.

**Figure 3:**
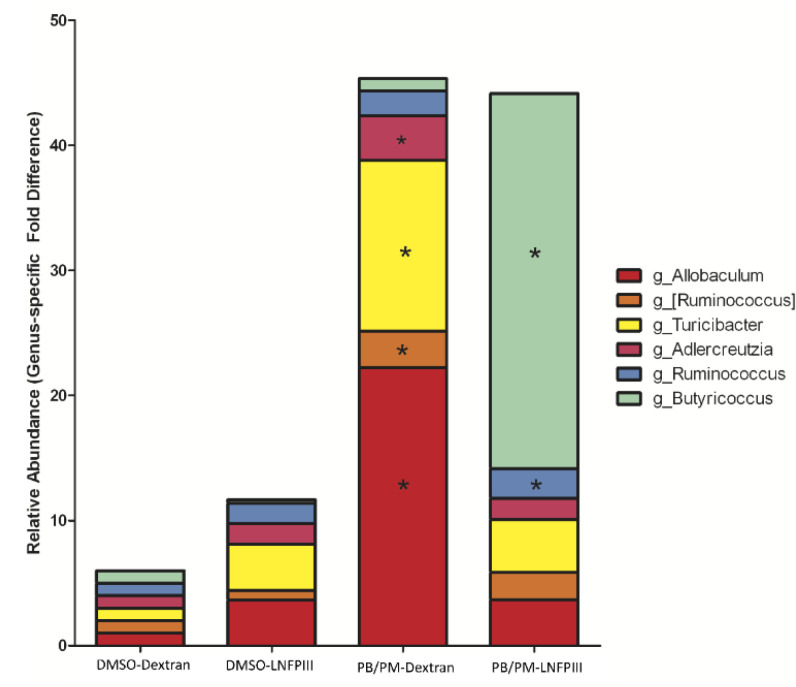
Genus-specific fold differences in relative sequence abundance for mice exposed either DMSO-LNFPIII, PB/PM-Dextran, or PB/PM-LNFPIII 9 months prior to sample collection relative to DMSO-Dextran control. (*) indicates statistical significance with respect to sequence abundance relative to all other treatment groups as determined using the Kruskall–Wallis one-way ANOVA on ranks (*p* < 0.05). Brackets indicate proposed relative bacterial abundance (OTUs) within the *Ruminococcus* genus (*Lachnospiraceae* family).

LEfSe is a method for determining organisms that are most likely to explain biological differences by applying non-parametric statistical tests taking into account effect size [51]. While PERMANOVA revealed no statistically significant effects of GWI chemical exposure or LNFPIII based on global microbiota composition, LEfSe uncovered specific taxa, most prominent in the chronic study samples, that are significantly affected. When all treatment groups from the 6 h samples were compared, only one bacterial family/genus was found to be affected by any treatment: a significant increase in the genus *Lactobacillus* within the PB/PM-Dextran group (Figure 2A). LEfSe did not identify any significantly affected bacteria at the 48 h time point. However, multiple taxa were significantly affected in the fecal samples 9 months post PB/PM treatment (Figure 2B). Numerous taxa were significantly increased in the DMSO-Dextran group, but the *Verrucomicrobiaceae* genus *Akkermansia* was the only one found to be increased in the DMSO-LNFPIII group (Figure 2B). Interestingly, the genus *Allobaculum*, within the family *Erysipelotrichaceae*, was significantly increased in the PB/PM-Dextran group, but the relative abundance of this genus in the PB/PM-LNFPIII group was similar to control levels (Figure 2B). Similar results were observed for the *Turicibacter* and *Adlercreutzia* genera, which are part of the families *Erysipelotrichaceae* and *Coriobacteriaceae*, respectively (Figure 2B). Of note, in PB/PM-LNFPIII mice, the genera *Ruminococcus* and *Butyricoccus* were significantly increased (Figure 2B). Figure 3 highlights the significant increase in the relative abundance of the genus *Allobaculum* in PB/PM-Dextran mice and the significant increase in the relative abundance of the genera *Ruminococcus* and *Butyricoccus* in mice exposed to PB/PM-LNFPIII when compared to all other treatments.

**Figure 4:**
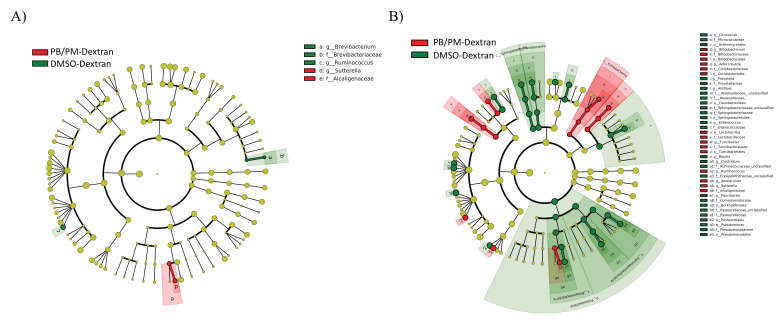
Linear discriminant analysis (LDA) effect size (LEfSe; Kruskall–Wallis (*p* < 0.05); Pairwise Wilcoxon (*p* < 0.05); logarithmic LDA score > 2.0) of the fecal microbiota of mice exposed to either DMSO-Dextran or PB/PM-Dextran. Taxa with increased sequence abundance after (**A**) 48 h or (**B**) 9 months post GWI exposures are indicated for PB/PM-Dextran (Red), DMSO-Dextran (Green) groups. Taxonomic rank labels are provided before bacterial names: “p_; c_; o_; f_; g_” indicate phylum, class, order, family, and genus, respectively. Letters and numbers within the cladograms refer to bacterial names located in the key to the right of each cladogram.

After comparing taxa between mice exposed to DMSO-Dextran and PB/PM-Dextran to assess effects of GWI-related chemicals in the absence of LNFPIII, no significant effects were found in the 6 h samples (Figure S2). At 48 h, PB/PM-Dextran exposure significantly increased the abundance of the family *Alcaligenaceae* and genus *Sutterella*, while it significantly decreased the genera *Brevibacterium* and *Ruminococcus* (Figure 4A). Most differences were observed in the samples from the chronic study. Thus, the *Adlercreutzia*, *Bifidobacterium*, *Ruminococcus*, *Allobaculum*, *Sutterella*, *Turicibacter*, and *Lactobacillus* genera were all significantly increased in GWI mice when compared to controls DMSO-Dextran), whereas the genera *Citricoccus*, *Prevotella*, *Alistipes*, *Enterococcus*, *Blautia*, *Clostridium*, *Paucibacter*, and *Pseudomonas* were decreased by PB/PM-Dextran (Figure 4B). Multiple other classes, orders, and families were influenced by GWI-related chemicals exposure (Figure 4B).

**Figure 5:**
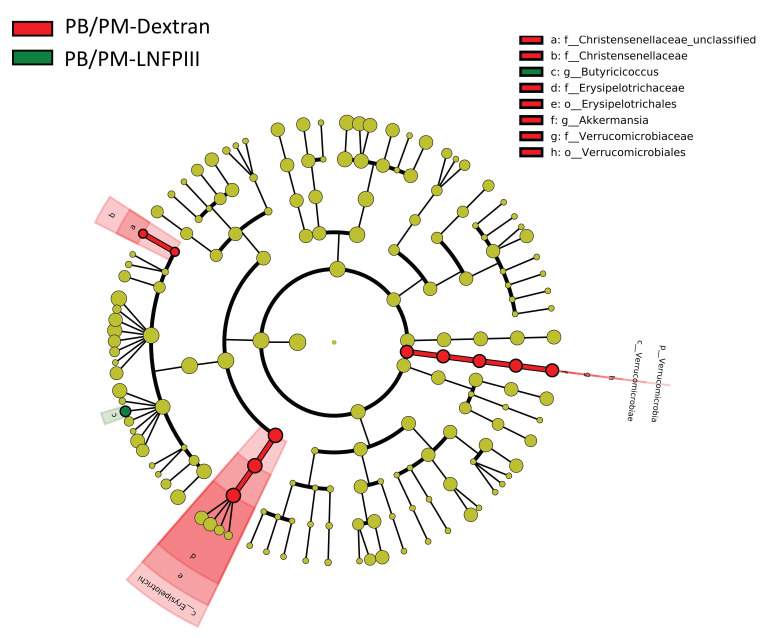
Linear discriminant analysis (LDA) effect size (LEfSe; Kruskall–Wallis (*p* < 0.05); Pairwise Wilcoxon (*p* < 0.05); logarithmic LDA score > 2.0) of the fecal microbiota of PB/PM-Dextran or PB/PM-LNFPIII mice collected 9 months after GWI exposures. Taxa with increased sequence abundances in either PB/PM-Dextran (Red) or PB/PM-LNFPIII (Green) are marked. Taxonomic rank labels are provided before bacterial names: “p_; c_; o_; f_; g_” indicate phylum, class, order, family, and genus, respectively. Letters within the cladogram refer to bacterial names located in the key to the right.

Interestingly, when LNFPIII effects were evaluated within PB/PM context in the chronic samples, LNFPIII administration led to a significant increase in the abundance *Butyricoccus* genus, which was decreased by PB/PM, and it prevented increases in the abundances of *Akkermansia*, *Christensenellaceae* and *Erysipelotrichaceae* (Figure 5).

### 3.3. Gut Motility

**Figure 6:**
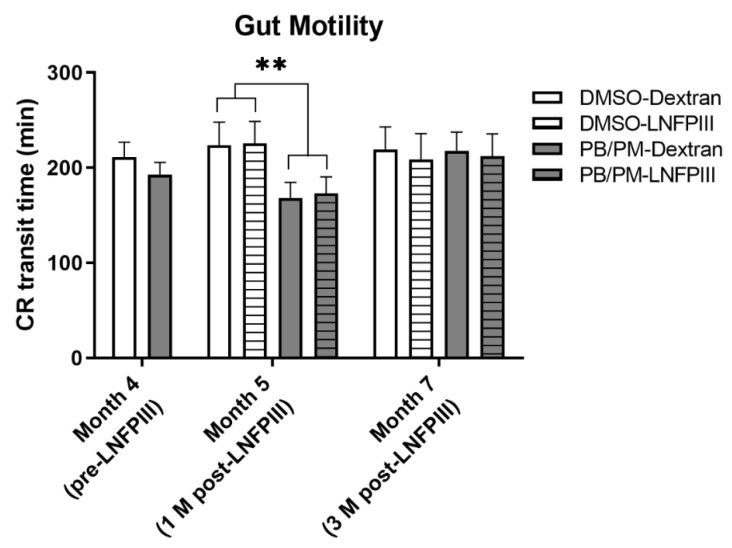
Bar plot showing gut transit time of carmine red (CR), 4, 5 or 7 months after mice were exposed to DMSO vehicle or PB/PM. Dextran vehicle or LNFPIII treatments started after CR testing at 4 months. Data are presented as mean ± SEM and sample sizes were *n* = 27–29/group at 4 months and *n* = 13–16 at months 5–7. (** *p* < 0.01).

There were no significant differences in carmine red gut transit time between DMSO and PB/PM groups 4 months post GW chemical exposure (Figure 6). One month later (month 5), after 1 month of LNFPIII treatment, there was a significant decrease in gut transit time (e.g., shorter transit time) for PB/PM groups (Figure 6; *p* ≤ 0.01), suggesting GI disruption. However, this was a transient effect, as no significant differences between treatments for transit time were present at 7 months (Figure 6) or for the overall average of the 3 months (months 5–7; data not shown) post LNFPIII treatment initiation.

### 3.4. Lipocalin-2 ELISA

**Figure 7:**
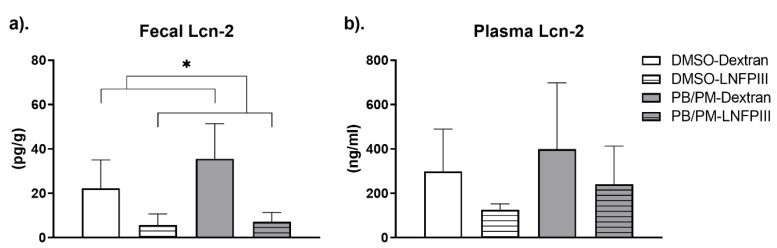
Bar plot showing levels of Lipocalin-2 (Lcn-2), as detected by ELISA, in mouse (**a**) fecal (pg/mL) and (**b**) plasma (ng/mL) samples from mice that were exposed to either DMSO-Dextran, DMSO-LNFPIII, PB/PM-Dextran, or PB/PM-LNFPIII and sacrificed 9 months later. Data are presented as mean ± SEM (*n* = 6/group). (* *p* < 0.05 post PB/PM treatment).

Fecal Lcn-2 levels at the end of the chronic study were numerically elevated by prior PB/PM exposure. LNFPIII treatment significantly decreased the levels of fecal Lcn-2 (Figure 7a; *p* ≤ 0.05) in both DMSO and PB/PM groups, suggesting an overall reduction in intestinal inflammation with this treatment. Similar trends, although not significant, were present in plasma Lcn-2 levels (Figure 7b).

### 3.5. Plasma Cytokines

There were no statistically significant differences among the plasma cytokines/chemokines examined. However, there were numerical increases in the PB/PM-Dextran group that were not observed in samples from mice treated with LNFPIII for IL-6, IL-15, IL-17A, IL-17F, and IL-22 (Figure S3). Further, there was a numeric decrease in IL-28b levels in the PB/PM treated groups compared to the DMSO groups (Figure S3). Several cytokines (i.e., IL-10, TNFα and TNFβ) were at or below the limit of detection.

### 3.6. Correlational Analysis between Bacterial OTUs and Behavioral Task/Lcn-2

**Figure 8:**
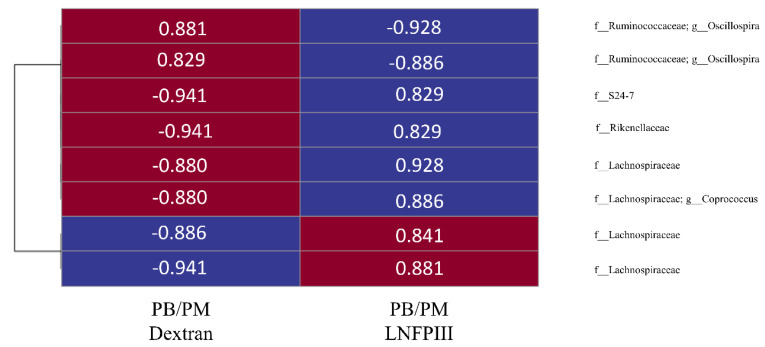
Heat map representing the relative bacterial abundance (OTUs) belonging to families specified to the right of the figure that were significantly (*p* < 0.05) correlated with the fastest sticker removal time (sec) in mice exposed to PB/PM-Dextran (left column) or PB/PM-LNFPIII (right column). White numerals indicate the correlation coefficient between relative abundance and fastest removal time for each respective OTU. The “f_” indicates family level and “g_” indicates genus level of taxonomic ranks. If no genus is provided, the OTU was unclassified at the genus level. Red boxes indicate increased and blue color indicate decreased relative abundance of each family/genus OTU in that respective treatment group (column). No sign in front of the number indicates it is a positive correlation coefficient, whereas a negative sign indicates a negative correlation coefficient.

A concurrent study focused on long-term neurological effects of PB/PM found that PB/PM treatment increased sticker removal time (a sensorimotor coordination test), and LNFPIII eliminated this effect. Using these data revealed eight OTUs (relative bacterial abundance) that significantly (*p* < 0.05) correlated, in both groups, with fastest sticker removal time (Figure 8). Of those OTUs with relative abundance higher in the PB/PM-Dextran mice, two, belonging to *Ruminococcaceae Oscillospira,* were positively correlated with fastest removal time in PB/PM-Dextran and negatively correlated in PB/PM-LNFPIII mice. In contrast, two *Lachnospiraceae*, one *Rikenellaceae*, and one candidate family *S24-7* OTUs were significantly negatively correlated in PB/PM-Dextran mice, but significantly positively correlated in PB/PM-LNFPIII mice (Figure 8). Of those OTUs with relative abundance higher in PB/PM-LNFPIII mice, two belonging to the *Lachnospiraceae* were significantly negatively correlated in PB/PM-Dextran and positively correlated in PB/PM-LNFPIII mice (Figure 8).

**Figure 9:**
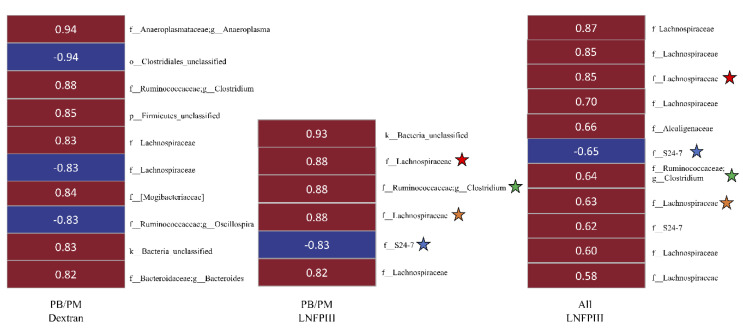
Heat map representing the relative abundance of OTUs that were significantly (*p* < 0.05) correlated with the fecal lipocalin-2 (Lcn-2) levels in mice exposed to PB/PM-Dextran (left column), PB/PM-LNFPIII (middle column), or collapsed LNFPIII groups (right column). White numerals display the correlation coefficient between relative abundance and fecal Lcn-2. Each OTU phylogenetic identification is provided to the right of each column. Stars (color-matched) designate OTUs that overlap between PB/PM-LNFPIII and collapsed LNFPIII treatment groups. Taxonomic rank labels are provided with “k_; p_; o_; f_; g_” indicating kingdom, phylum, order, family, and genus, respectively. If no genus is provided, the OTU was unclassified at the genus level. Here, red and blue boxes indicated positive or negative correlation coefficients, respectively, with the exact correlation coefficient within each treatment being listed within the respective box.

Next, significant correlations (*p* < 0.05) were investigated between bacterial OTUs and fecal Lcn-2 levels. Unlike the correlations with sticker removal performance, there were no OTUs that significantly correlated with fecal Lcn-2 in both PB/PM-dextran and PB/PM-LNFPIII treatment groups. However, numerous OTUs correlated to Lcn-2 within groups (Figure 9). For example, within the PB/PM-Dextran group, OTUs belonging to *Anaeroplasma*, *Clostridium*, and *Bacteroides*, along with OTUs within the families *Lachnospiraceae*/*Mogibacteriaceae* were significantly positively correlated with fecal Lcn-2. In contrast, one *Lachnospiraceae*, one *Oscillospira* (family *Ruminococcaceae*) and one unclassified *Clostridiales* OTU were all negatively correlated (Figure 9). In the PB/PM-LNFPIII group, three *Lachnospiraceae*, one *Clostridium* (*Ruminococcaceae*), and one unclassified OTUs were all significantly positively correlated, whereas one candidate family *S24-7* OTU was significantly negatively correlated with fecal Lcn-2 (Figure 9). Correlations between OTUs and Lcn-2 in all animals that received LNFPIII (i.e., both DMSO-LNFPIII and PB/PM-LNFPIII) were also assessed; ten and one OTUs were significantly positively and negatively correlated, respectively (Figure 9). Similar to the PB/PM-LNFPIII analysis, only one candidate family *S24-7* OTU was negatively correlated with Lcn-2. Seven *Lachnospiraceae*, one *Alcaligenaceae*, one *Ruminococcaceae*, and one candidate family *S24-7* OTUs were significantly positively correlated with Lcn-2 in all LNFPIII mice (Figure 9). Of note, four identical OTUs, belonging to the *Lachnospiraceae* (2), *Clostridium* (1)*,* and *S24-7* (1), correlated with fecal Lcn-2 within the PB/PM-LNFPIII and across both LNFPIII-treated groups (Figure 9).

## 4. Discussion

Recent evidence suggests that the enteric microbiome, in conjunction with widespread immunological perturbations, may play a significant role in GWI symptomology, as well as provide novel therapeutic targets for veterans with this chronic illness. This study sought to evaluate microbiota perturbations in mice exposed to two chemicals that are epidemiologically associated with GWI development and used in an established model of GWI [18]. The second goal of this study was to assess whether an immunomodulatory glycan, LNFPIII, could potentially provide therapeutic benefit through the complex interaction between the microbiota, immune system, and physiological homeostasis. While exposure to PB/PM and/or LNFPIII did not significantly impact the species composition of the microbiota on a global scale, this study found that both PB/PM and LNFPIII resulted in the enrichment/depletion of specific bacterial taxa, and that some bacterial OTUs were significantly correlated with GWI (patho)physiological endpoints of interest.

There were no significant effects of exposure to GWI-related chemicals or LNFPIII on either alpha diversity metric considered (i.e., Chao1 richness or Shannon’s diversity index), with the exception of the chronic study where Shannon’s diversity was increased slightly by PB/PM. Notably, LNFPIII treatment skewed diversity towards control levels. A recent study in a GWI mouse model, that is based on the same GWI chemicals used in the current study, but also includes stress, reported significant increases in both richness and diversity [10]. Veterans with GWI, regardless of GI symptom presence, had significantly higher sample richness and numerically lower Shannon’s diversity in a small preliminary study [11]. Other studies focused on the effects of GWI on the enteric microbiota and/or putative therapeutic interventions did not report diversity analysis. Overall, the data herein aligns with previous studies in mouse models of GWI. The data on alpha diversity in GWI veterans might need to be stratified by exposure severity and/or type to have better concordance between rodent models and GWI veterans.

Importantly, this study found significant perturbations of specific bacterial taxa. Notably, *Allobaculum* was increased in the PB/PM-Dextran group, with LNFPIII administration returning *Allobaculum* abundance to near control levels. This genus was previously found to be significantly increased in another mouse model of GWI [10] and has been shown to have increased abundance in rats on a high-cholesterol diet alongside a negative correlation with colonic IL-10 and Foxp3 mRNA expression [55]. Interestingly, only Foxp3 was significantly decreased in the high-cholesterol group and the negative correlation coefficient between *Allobaculum* and Foxp3 was stronger than with IL-10. Considering that LNFPIII attenuated *Allobaculum* increases in mice exposed to GWI chemicals, the relationship between *Allobaculum* spp., GWI-related exposures, and enteric Foxp3, or other anti-inflammatory genes, is worthy of investigation. How *Allobaculum* affects volatile fatty acids (VFA) status, a key indicator of gut health, is up for debate and subject to investigation. While one study investigating a novel bacterium, *Allobaculum stercoricanis*, in canine feces found that butyrate is one by-product of glucose metabolism [56], another study of *Allobaculum* spp. in the murine microbiota found that lactate, acetate, and propionate are prominent glucose metabolites [57]. Overall, while consistent increases in the *Allobaculum* genus have been identified by us and [10], further interrogation of the *Allobaculum* species/strains may provide greater insights into the role *Allobaculum* changes may play in promotion/attenuation of GWI-related symptoms, including by promoting favorable VFA profile, i.e., increased butyrate.

One recent study investigating the interaction between the microbiota, enteric/systemic inflammation, and GWI found that butyrate priming might be therapeutically valuable after finding GWI models exhibited statistically significant decreases in *Bifidobacterium* and *Lactobacillus* [12]. While this previous study did not provide pertinent details about how GWI treatment effected the microbiota prior to butyrate administration, they found that butyrate priming in a GWI mouse model resulted in a slight increase in the abundance of bacteria with the genera *Bifidobacterium*, *Lactobacillus*, and *Roseburia*, as well as recovery from leaky gut syndrome and other metabolic indicators [12]. While these results may have beneficial implications for future GWI therapeutics, the bacteria identified do not utilize butyrate as a metabolic substrate and the main VFA produced by *Lactobacillus* is lactate. Thus, the connection between butyrate priming and recovery of pathophysiological signs is likely a result of increased butyrate bioavailability along the enteric tract from exogenous administration. Moreover, while identifying bacterial taxa at the genus level can be a beneficial step towards developing beneficial probiotics, blanket generalization of certain genera may have mixed effects, as is the case for bacteria in the genera *Bifidobacterium* [25,26,27,28,29,30] and *Roseburia* [58]. However, it is plausible that increasing the abundance of butyrate producing bacteria is one potential therapeutic route to alleviate GI and, perhaps other, symptoms in veterans with GWI [59]. In this light, data provided herein is noteworthy as LNFPIII treatment increased the *Ruminococcus* and *Butyricoccus* genera, indicating this immunomodulatory glycan may influence the microbiota in a manner that enables increased butyrate production. Although we did not measure this critical, or other, VFAs, it would be important that future work evaluates the abundance of VFAs within the enteric tract, while, concomitantly, performs deep sequencing of these two genera; this will delineate specific species and/or strain-specific effects that might be beneficial for alleviating GWI symptomology.

This study was unable to identify persisting, significant effects of either GWI or LNFPIII on gut motility after 4 months of exposure to GWI chemicals. A significant decrease in gut transit time, suggestive of GI disruption, was present in PB/PM groups at month 5. However, this effect was transient, as it was not observed at any other timepoints. While effects of GWI on enteric inflammation and physiology have been previously identified [10,12], only one other study assessed gut motility and found that exposure to PB caused acute and chronic alterations in gut motility [13]. On the other hand, previous studies have consistently found exposures associated with GWI result in impairments in gut wall integrity and alterations of the enteric inflammatory profile [10,11,12,13]. Herein, fecal and plasma Lcn-2 (NGAL), a protein that has been shown to elicit intestinal inflammation and is a good biomarker of intestinal inflammation [60], was numerically increased in mice exposed to PB/PM, when compared to controls. Further, LNFPIII decreased both fecal (significantly) and plasma (trend) Lcn-2 levels when compared to either set of controls, indicating a potential beneficial role for LNFPIII in reducing intestinal inflammation not only in GWI, but also in normal aging. The mice in the chronic study were almost a year old at the time of fecal sample collection for Lcn-2. Aging is consistently associated with increases in GI inflammation [61,62]. The fact that LNFPIII also decreased fecal Lcn-2 in control mice suggests an added benefit of this molecule in preventing age-related gut inflammation. Although the levels of circulating plasma cytokines were mostly unaffected by either treatment, numerical increases in inflammatory cytokines such as IL-6, IL-15, and IL-17 F by PB/PM were observed. These peripheral inflammatory increases were not apparent in LNFPIII groups, and this treatment had a further modulating impact on inflammatory IL-17A and IL-22 levels. While it is unclear whether exposure to the PB/PM mixture can negate the effects of PB alone on gut motility [13], the influence of GWI-related chemicals on intestinal inflammation in this study is consistent to a previous report [12], but perhaps less pronounced due to the nature of the model and/or timing of assessment.

Another point of recent interest in the larger microbiome community has been the interaction between the gut microbiota, neuroinflammation, and alterations in motor behavior, namely motor deficits. This interest is derived from the suggested use of primary GI symptoms as early biomarkers of potential neurodegenerative diseases, such as PD [7]. In fact, in addition to the beneficial effects of butyrate in the gut, studies have indicated that increases in butyrate improve neurobehavioral function including cognitive, mood, and motor functions in numerous settings [63,64,65,66]. Considering the relationship between GWI, motor deficits and PD, this study sought to identify preliminary associations between the hindgut microbiota and a sensorimotor task (sticker removal). This sensorimotor task requires a mouse to detect and have the ability to remove a sticker placed on its snout, with increased latency to contact and removal of the sticker indicating a motor deficit [67]. Behavioral data in mice treated with PB/PM indicated a deficiency in this task, which LNFPIII treatment prevented (unpublished). Eight OTUs (two *Ruminococcaceae*, four *Lachnospiraceae*, one *Rikenellaceae*, and one candidate *S24-7*) were identified that significantly correlated with the fastest sticker removal time in both PB/PM-Dextran and PB/PM-LNFPIII. Of note, the correlation coefficients of these OTUs were opposite between the two experimental treatment groups for all OTUs, indicating some interplay may exist between these bacteria, GWI-related exposures, LNFPIII modulation, and fine motor tasks. Although this will require further investigation, this is an important starting point moving forward for understanding the interplay of these factors and the complexity of GWI, with the ultimate goal of providing relief to veterans experiencing GWI from GI, neurological, and perhaps other symptoms.

Finally, this study identified numerous OTUs that were significantly correlated with fecal Lcn-2 and these OTUs were different across treatments. While most OTUs identified were from the predominant families (i.e., *Lachnospiraceae* and *Ruminococcaceae)* and have been previously reported in GWI context [11], it is worth noting that some have previously been correlated with symptoms of IBS as well [68]. Notably, there were a number of OTUs from mice treated with LNFPIII which correlated with fecal Lcn-2; several of them were correlated with Lcn-2 regardless of PB/PM treatment. Given that LNFPIII decreased fecal Lcn-2 irrespective of GWI treatment, future studies further investigating the modulatory role that these bacteria play in intestinal inflammation are warranted.

## 5. Conclusions

This study provides additional evidence to support previous work showing that GWI-related chemical exposure induces significant perturbations of select bacterial taxa of the mouse GI microbiota. Additionally, LNFPIII treatment mitigated increases in certain bacteria (e.g., *Allobaculum*) thought to be associated with pathophysiological outcomes of interest in GWI mouse models. Administration of LNFPIII concomitantly resulted in an increase in specific genera that include species known to produce butyrate, a beneficial VFA, thought to have potential multi-factorial benefits in a GWI context. These benefits extend to altering gut health by reducing gut inflammation produced by prior GWI exposure and aging. Overall, this work provides additional support for moving forward and investigating the complex interactions between the microbiome/GI health, immune and nervous systems, under the umbrella of GWI, while providing additional information about how immunomodulatory compounds (e.g., LNFPIII) could provide multi-level benefits for GWI symptom management. In addition to more mechanistic studies, it will be important to integrate these findings in clinical studies where LNFPIII’s benefits are evaluated in veterans with GWI.

## Supplementary Materials

The following are available online at https://www.mdpi.com/1660-4601/17/19/7081/s1, Figure S1: Chao1 richness and inverse of Simpson’s diversity index in the long-term GWI study, Figure S2: LEfSe 6 h post GWI exposures, Figure S3: Heat map of selected plasma cytokines at the end of the long-term GWI study.
