# Supplementary material for: Assessing the Beneficial Effects of the Immunomodulatory Glycan LNFPIII on Gut Microbiota and Health in a Mouse Model of Gulf War Illness
Source: Int J Environ Res Public Health. 2020 Sep 27;17(19):7081. doi: 10.3390/ijerph17197081 (PMC7579323; doi:10.3390/ijerph17197081)
Supplement: Supplementary file 1 [file ijerph-17-07081-s001.pdf]

Supplementary Materials: The following are available online at www.mdpi.com/xxx, Figure S1: title, Table S1: title, Video S1: title.

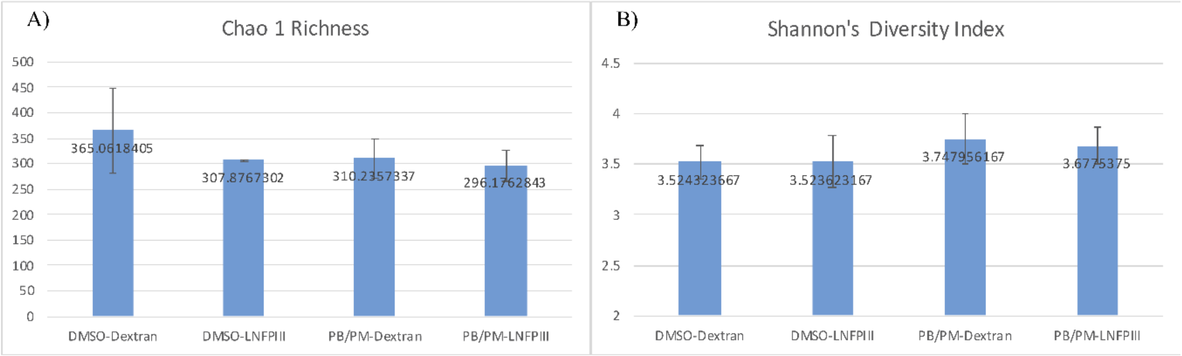

Figure S1: Changes in (A) richness (Chao1 richness) and (B) diversity (inverse of Simpson's diversity index) in the mice exposed to either DMSO-Dextran, DMSO-LNFPIII, PB/PM-Dextran, and PB/PM-LNFPIII in the long-term (chronic) study.

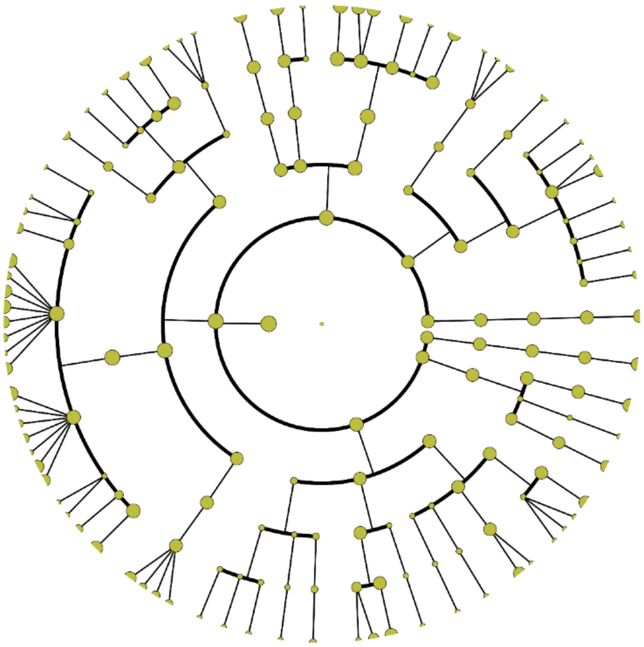

Figure S2. Linear discriminant analysis (LDA) effect size (LEfSe; Kruskall-Wallis [P < 0.05]; Pairwise Wilcoxon [P < 0.05]; logarithmic LDA score > 2.0) of the fecal microbiota of mice exposed to either DMSO-Dextran or PB/PM-Dextran after 6 h post GWI exposures. As no taxa were altered, no groups are highlighted and no labels are provided.

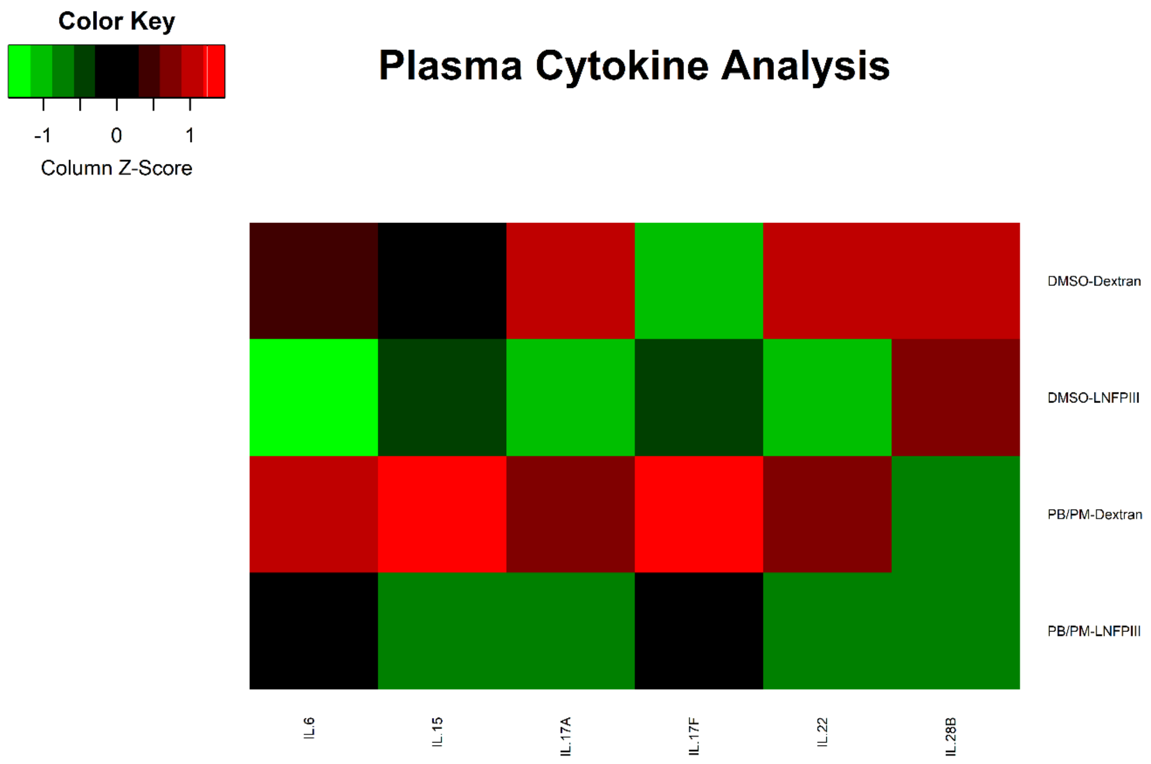

Figure S3. **Heat map of selected plasma cytokines.** Heat map representing selected cytokines in mice exposed to DMSO-Dextran (n=6), DMSO-LNFPIII (n=6), PB/PM-Dextran (n=7), and PB/PM-LNFPIII (n=7). Red and green color indicates increases and decreases, respectively, of plasma cytokine levels. Color key with Z-scores is located on the upper left.
